# Clinical Features and Surgical Treatment of Subretinal Proliferation in Proliferative Diabetic Retinopathy

**DOI:** 10.3389/fmed.2022.833519

**Published:** 2022-02-11

**Authors:** Chan Wu, Rongping Dai, Youxin Chen, Xiao Zhang, Zhe Chen

**Affiliations:** ^1^Department of Ophthalmology, Peking Union Medical College Hospital, Peking Union Medical College, Chinese Academy of Medical Sciences, Beijing, China; ^2^Key Lab of Ocular Fundus Diseases, Chinese Academy of Medical Sciences, Beijing, China

**Keywords:** subretinal proliferation (SRP), subretinal band, proliferative diabetic retinopathy (PDR), tractional retinal detachment (TRD), vitrectomy

## Abstract

**Purpose:**

To describe the clinical characteristics, treatments, and prognosis of subretinal proliferation (SRP) in proliferative diabetic retinopathy (PDR) patients.

**Methods:**

A total of 184 patients (221 eyes) who received vitrectomy for PDR between 2018 and 2021 were retrospectively reviewed. Patients with SRP were further evaluated. The following data were collected from their medical records: demographics, systemic and ophthalmologic findings, and treatment given specifically for SRP. The main outcome measures included the visual acuity (VA), funduscopic examination, and final anatomic success.

**Results:**

Twelve eyes of eleven patients including seven females and four males with a mean age of 47.64 ± 11.21 years were evaluated. The surgical indication for the patients was mainly tractional retinal detachment (TRD) (100.0%). No retinal break was found preoperatively or intraoperatively. Only one eye (8.3%) had undergone subretinal band removal procedure intraoperatively, and the final anatomical success rate was 100%.

**Conclusion:**

Subretinal proliferation in PDR was associated with TRD. The retina could reattach successfully after vitrectomy without removal or transection of SRP in most eyes.

## Introduction

Proliferative diabetic retinopathy (PDR) is one of the leading causes of blindness worldwide (1). It is characterized by neovascularization originating from the retina and optic disc as a severe complication of diabetes mellitus (DM). The new vessels often grow along the vitreoretinal interface and sometimes into the vitreous, leading to vitreous hemorrhage (VH), epiretinal fibrovascular membranes (FVMs), and subsequent tractional or combined tractional-rhegmatogenous retinal detachment, for which surgical intervention is indicated to avoid severe vision loss (2). The formation of epiretinal FVMs is a proliferative process on the inner surface of the retina, which is frequently found in PDR, whereas proliferation on the retroretinal surface is less common (3). Subretinal proliferation (SRP) is also known as subretinal band, subretinal strand, subretinal membrane, retroretinal membrane, or subretinal fibrosis. Subretinal proliferation occurs with long-standing exudative, traction, or rhegmatogenous retinal detachments but is most common in eyes with rhegmatogenous retinal detachment (RRD) complicated by proliferative vitreoretinopathy (PVR) (4, 5). Several studies have reported the pathogenesis, clinical features and surgical management of SRP in PVR (6–10). However, to the best of our knowledge, SRP in PDR has not been described in the literature. Therefore, the purpose of this study was to describe the clinical characteristics of SRP in PDR and to investigate its clinical treatments and prognosis.

## Materials and Methods

### Patients and Study Design

This retrospective observational study was approved by the Institutional Review Board (IRB) of Peking Union Medical College Hospital. All data with patient-specific information were masked and deidentified prior to analysis. Written informed consent was not required by the IRB but participants who did not grant authorization to use their medical records for the research were excluded from analyses. This study was performed according to the tenets of the Declaration of Helsinki.

We reviewed ophthalmological records of PDR patients who were treated with pars plana vitrectomy (PPV) in Peking Union Medical College Hospital by one experienced surgeon (RPD) between January 1, 2018 and October 31, 2021. All patients received vitrectomy because of reduced VA principally from non-clearing VH, fibrovascular proliferation affecting the macula, or tractional retinal detachment (TRD). Among all these PDR patients, those who presented with SRP were enrolled in the study. Exclusion criteria included (1) previous eye diseases such as RRD, uveitis, endophthalmitis, choroidal melanoma, Coats disease, or penetrating ocular trauma; (2) prior intraocular surgery such as cataract surgery, glaucoma surgery, vitrectomy, or scleral buckling.

### Surgical Methods

All patients underwent standard 23-gauge or 25-gauge three-port PPV with the Constellation Vitrectomy System (Alcon Labs, Fort Worth, TX, USA) under retrobulbar or general anesthesia. Procedures such as FVM dissection, endolaser photocoagulation, drainage of the subretinal fluid (SRF) through an artificial retinal hole, and air-fluid exchange were performed as needed. The decision to remove the subretinal proliferation was made intraoperatively if the subretinal tissue prevented proper flattening of the retina or if it caused distortion of the macula after drainage of the SRF. The subretinal band was removed by making a small incision in the detached retina and grasping the band with special forceps. Retinal tamponade was performed with gas or silicone oil as required. The silicone oil was removed 3–6 months after the initial procedure to avoid complications such as emulsification or secondary ocular hypertension. In patients with cataract, phacoemulsification with intraocular lens (IOL) implantation was performed immediately before vitrectomy or silicone oil removal surgery. No additional buckling was performed in any of our cases.

### Collections of Clinical Data

The demographic and clinical information (such as age, gender, and ocular and systemic examination results) of the included patients was extracted from medical records. Ophthalmological data collected from the charts included the study eye, presence of VH, presence and extent of retinal detachment (RD), presence of retinal breaks, presence of epiretinal FVMs, characteristics of SRP, prior panretinal photocoagulation (PRP), or intravitreal injection (IVI) of anti-VEGF drugs, peeling of epiretinal FVMs, removal or transection of SRP, gas or silicone oil infusion, anatomical outcome of surgery, and pre- and postoperative best corrected visual acuity (BCVA). Anatomical success was defined as total retinal reattachment after vitrectomy without silicone oil tamponade or after silicone-oil-removal surgery. Patients were followed every 3 months until the ocular condition was stable after the final surgery.

### Statistical Analysis

Statistical analyses were carried out using the SPSS software package, V.22.0. Quantitative data were presented as mean ± SD for parametric data. For statistical analysis, VA levels for individual patients reported as the Snellen acuity or decimal acuity scores were converted to logarithm of the minimum angle of resolution (logMAR) scores using standard calculations (11). The LogMAR values of 2.0, 2.4, 2.7, and 3.0 were substituted for VA levels reported as “count fingers,” “hand movements,” “light perception,” and “no light perception,” respectively (12). The mean VA (and standard deviation) at baseline and follow-up and changes in VA were calculated using the logMAR scores.

## Results

### Demographic and Systemic Findings

Among 221 eyes of 184 PDR patients evaluated, 12 eyes of 11 patients were diagnosed with SRP and were further evaluated. The overall prevalence of SRP was 5.43%. The average age of the patients was 47.64 ± 11.21 years. There were seven female (63.6%) and four male patients. The mean duration from the diagnosis of DM to operation was 11.00 ± 7.46 years. The average fasting blood glucose (FBG) was 11.51 ± 3.17 mmol/L on presentation. There were four patients (36.4%) had hypertension, two patients (18.2%) had cardiovascular disease, and three patients (27.3%) had renal disease. The average creatinine level was 65.36 ± 33.99 μmol/L, while the average fibrinogen level was 3.67 ± 0.61 g/L.

### Preoperative and Intraoperative Findings

**Figure 1:**
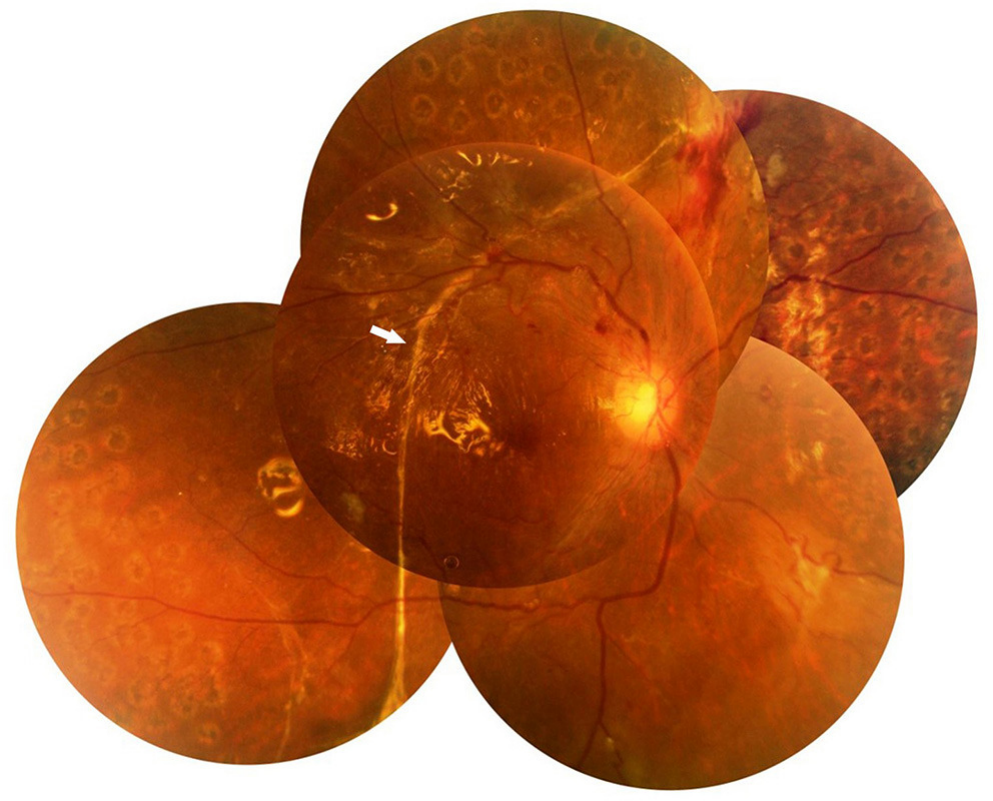
Fundus photograph of case 3. It shows a well-attached retina and temporal long linear subretinal proliferation (SRP) (arrow) 1 month after pars plana vitrectomy (PPV).

**Figure 2:**
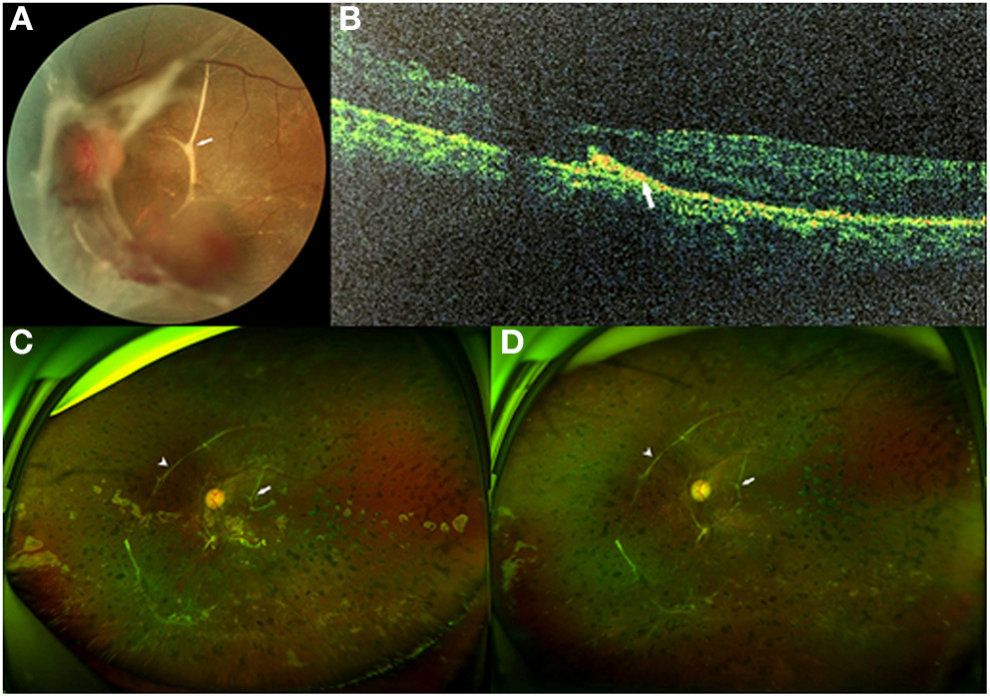
Fundus photographs of case 5. **(A)** Preoperative fundus photograph showing tractional retinal detachment (TRD) from the para-inferotemporal arcade to nasal area with epiretinal fibrovascular membranes (FVMs) and submacular branching band (arrow) in the left eye. **(B)** Preoperative spectral domain-optical coherence tomography (SD-OCT) demonstrated a hyperreflective subretinal band (arrow) with thinning of the outer retina. **(C)** Three months after pars plana vitrectomy (PPV) with silicone oil tamponade: ultrawide-field fundus photograph showing the attached retina with a branching subretinal proliferation (SRP) near the macula and papilla (arrow), and a long linear SRP at the nasal area (arrowhead), which had already existed before the surgery. **(D)** Three months after silicone oil removal surgery: ultrawide-field fundus photograph still showing the attached retina with a long, linear SRP at the nasal area (arrowhead) and a branching SRP near the macula and papilla (arrow).

**Figure 3:**
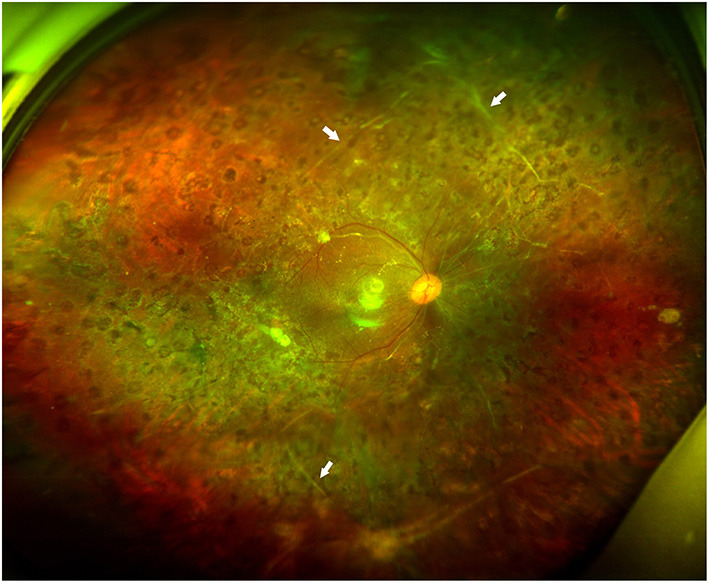
Fundus photographs of case 9. It shows a well-attached retina without subretinal band (arrows) removal 3 months after silicone oil removal surgery.

Among the twelve eyes that had SRP, six were right eyes and six were left eyes. Prior IV-anti-VEGF was not performed in any eye. Panretinal photocoagulation was performed in two eyes. Six eyes had VH, while all twelve eyes had TRD. The surgical indication for the patients was mainly TRD (100.0%). The mean LogMAR of BCVA before the surgery was 1.41 ± 0.48. Epiretinal FVMs were detected in all eyes. No retinal break was detected in any eye. Retinal detachment involved one quadrant in three eyes (25.0%), two quadrants in three eyes (25.0%), three quadrants in two eyes (16.7%), and total RD was found in four eyes (33.3%). Eight eyes had only one subretinal band (66.7%) (Figure 1), while two eyes had two subretinal bands (16.7%) (Figure 2), and two eyes had three subretinal bands (16.7%) (Figure 3). Ten eyes had linear-shaped bands (83.3%), which were mainly located in the midperiphery and parallel to the borderline between the detached and unaffected retina. One eye had a band with many branches (8.3%), and one eye had both linear-shaped and branching bands (8.3%) (Figure 2). Both of the branching bands were located at the posterior pole; one was right beneath the macula, and one was near the optic disc.

### Details of Operation

**Figure 4:**
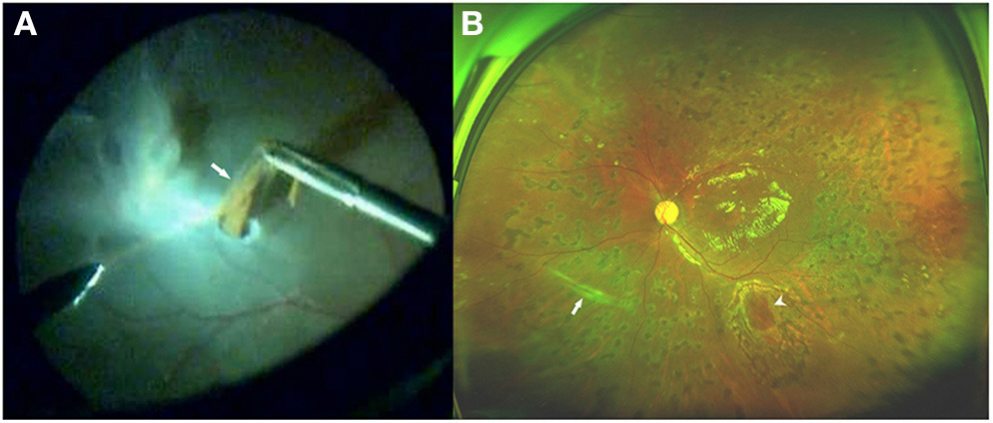
Fundus photograph of case 11. **(A)** Intraoperative photo showing the removal of the inferonasal subretinal proliferation (SRP) (arrow) through a small incision in the detached retina. Note that the SRP contains many pigments. **(B)** Three months after pars plana vitrectomy (PPV), ultrawide-field fundus photograph demonstrating a well-attached retina, and a trace of inferonasal linear SRP (arrow), which had been removed during the vitrectomy. The edge of the incision (arrowhead) is seen in the inferior area.

Due to the lack of inert gases during that period in China, we performed PPV with silicone oil tamponade in 100% of the cases because most of the cases were severe. Only one eye (8.3%) had undergone subretinal band removal or transection procedure intraoperatively (Figure 4). Silicone oil was removed 3–6 months after the initial vitrectomy in all eyes. Phacoemulsification with IOL implantation was performed simultaneously during the silicone-oil-removal surgery in four eyes (33.3%). The detailed examination data and operative methods are listed in Table 1.

**Table 1:** Characteristics of proliferative diabetic retinopathy (PDR) with subretinal proliferation (SRP).

**Pt no**.	**Gender**	**Age (yr)**	**Laterality**	**Baseline BCVA**	**VH**	**Extent of RD**	**Extent of SRP**	**Shape of SRP**	**Retinal break**	**Prior IVI anti-VEGF**	**Prior PRP**	**Removal of SRP**	**Intra-OP SO**	**BCVA after PPV (1 w)**	**PHACO during SO removal**	**Final BCVA**	^*^Follow-up duration (m)
1	F	53	OD	HM	N	Total	Inferotemporal	Linear	N	N	N	N	Y	CF	N	20/200	6
2	M	60	OS	20/250	Y	Inferior and temporal	Inferotemporal	Linear	N	N	Y	N	Y	HM	Y	20/1,000	12
3	F	30	OD	20/500	N	Total	Temporal	Linear	N	N	N	N	Y	20/500	N	20/100	18
4	M	67	OD	20/100	N	Superior, inferior and nasal	Inferonasal	Linear	N	N	N	N	Y	20/1,000	Y	20/250	6
5	F	37	OS	20/2,000	Y	Nasal and para-inferotemporal arcade	Macular and nasal	Branched and linear	N	N	N	N	Y	CF	N	20/1,000	12
6	F	49	OD	20/1,000	N	Posterior	Peripapillary	Branched	N	N	N	N	Y	HM	N	20/250	9
7	F	52	OS	20/400	N	Total	Superior, inferior and nasal	Linear	N	N	N	N	Y	HM	N	20/200	9
8	F	43	OS	20/400	Y	Inferior and temporal	Inferior	Linear	N	N	Y	N	Y	20/1,000	N	20/66	18
9	M	52	OD	20/1,000	N	Superior, nasal and inferior	Superior, superionasal and inferior	Linear	N	N	N	N	Y	CF	Y	20/100	12
10	M	52	OS	20/100	Y	Posterior	Inferonasal	Linear	N	N	N	N	Y	CF	Y	20/200	9
11	M	48	OS	20/200	Y	Total	Inferonasal	Linear	N	N	N	Y	Y	20/250	N	20/66	12
12	F	33	OD	10/1,000	Y	Posterior	Superior and nasal	Linear	N	N	N	N	Y	CF	N	20/66	9

*BCVA, best corrected visual acuity; CF, counting fingers; F, female; HM, hand movement; IVI, intravitreal injection; m, months; M, male; N, no; OCT, optical coherence tomography; OD, right eye; OP, operation; OS left eye; PHACO, phacoemulsification; PPV, pars plana vitrectomy; PRP, panretinal photocoagulation; Pt, patient; RD, retinal detachment; SO, silicone oil; SRP, subretinal proliferation; VA, visual acuity; VEGF, vascular endothelial growth factors; VH, vitreous hemorrhage; w, week; Y, yes; yr, years old*.

* *The follow-up duration was the follow-up length after the silicone oil removal surgery*.

### Clinical Outcomes After Operation

All cases had a follow-up of at least 6 months after the silicone-oil-removal surgery. The median time of follow-up was 10.5 months, ranging from 6 to 18 months. All eyes achieved reattachment after the silicone-oil-removal surgery without any severe complications (Figures 2, 3). The mean LogMAR of BCVA was 0.96 ± 0.42 6 months after the silicone-oil-removal surgery. The characteristics of these patients are showed in Table 1.

## Discussion

Subretinal proliferation is mainly known as a complication of RRD and is part of the spectrum of PVR. It has been reported in 3–15.5% of eyes with uncomplicated RRD and in 47% of eyes with RRD associated with PVR (4, 5, 13). Subretinal proliferation may also occur in several uveitis syndromes (14, 15), choroidal melanoma (16), and Coats disease (17). However, to the best of our knowledge, our study is the first to report SRP in PDR.

Although subretinal fibrosis in diabetic macular edema (DME) has been reported in the literature before (18), that fibrosis is completely different from the SRP we reported in the present study. Subretinal fibrosis is an elevated mound or a flat sheet of gray or white tissue located deep to the retina at or near the center of the macula. Subretinal fibrosis always develops after very severe, hard exudate, and has no association with RD (18). Clinicopathologic studies have shown that the accumulation of hard exudate in the outer retina in juxtaposition to the retinal pigment epithelium (RPE) is associated with focal metaplasia of the RPE, leading to fibrotic scar formation. The optical coherence tomography (OCT) findings revealed that the fibrosis appears to have replaced at least a portion of the outer retina, suggesting that the fibrosis may be “intraretinal” rather than “subretinal” (19).

In our study, all PDR patients with SRP had epiretinal FVMs and TRD. We did not find any retinal break preoperatively or intraoperatively. Our observation suggested that RRD was not likely the main mechanism in PDR patients with SRP. In RRD, liquefied vitreous flows through the retinal breaks into the subretinal space and forms SRF. Subretinal fluid may result in breakdown of the blood-retina barrier, and some serum components that stimulate the proliferation of RPE cells and retinal glial cells may penetrate the blood-ocular barrier, such as fibronectin, platelet-derived growth factor (PDGF), fibroblast growth factor (FGF), epidermal growth factor (EGF), glial maturation factor, glial growth factor, IL-1, glial growth-promoting peptides, and thrombin (20). In addition, once the neural retina has separated from the RPE, increased distance to the choroidal blood supply and reduced oxygen flux from the choroid to the inner segments lead to a loss of photoreceptor outer segments and, furthermore, to a proliferation of glial cells (21). Ultimately, SRP develops. In our study, though all PDR patients with SRP had non-RRD, they may have had a similar pathogenesis. The blood-retina barrier could be very poor in these PDR patients. Following the advance of TRD, long-standing accumulation of the SRF, which also contains some growth factors, could continuously stimulate RPE cells and glial cells, leading to SRP.

In a previous study, RRD of long duration, atrophic retinal breaks, young age, and greater number of detached quadrants were identified as factors significantly associated with SRP (5). Wallyn and Hilton reported that the incidence of SRP was associated with the duration of RRD, ranging from 0.8% in cases with a duration <1 month to 22% in cases with more than 2 years (13). In our investigation, all the PDR patients with SRP had TRD while only half of them had VH. Furthermore, the severity of VH in these cases was relatively mild and stale. These results may be explained by the longer course of PDR and more advanced PDR in these patients, which led to SRP following long-term TRD. No eye received prior IV-anti-VEGF and only two eyes received prior PRP, which suggest that the SRP in PDR had no association with anti-VEGF injection or PRP.

We observed that 83.3% of the eyes had only linear-shaped subretinal bands. Previous laboratory works have demonstrated that most cells exhibit polarity. Cells need a surface to grow on, and they usually settle onto a surface with a specific polarity (22). The subretinal space is a hostile environment for cells to proliferate under normal conditions. After detachment, the subretinal space converts into a tissue culture system, where the RPE cell-derived macrophages originally settle onto the back surface of the retina and then multiply (23). Some cells transform into fibroblasts, which in turn produce collagen. This process creates a surface that can be populated by additional cells (24, 25). As the cells proliferate around this material, they form a circular pattern and a linear band. The glial cells undergo a similar process. They can transform into myofibrocytes after long-standing RD. Once stabilized, the attached cells also begin to synthesize collagen and create the proliferation surface (26, 27). Finally, the linear-shaped band forms. In our study, the linear-shaped subretinal bands were mainly located in the midperiphery, and the direction of the subretinal bands was parallel to the borderline between the detached and unaffected retina. This finding may indicate that the RPE-derived cells and glial cells that compose the subretinal bands tend to accumulate along the borderline and proliferate. We also found that 16.6% of the eyes had branching subretinal bands, both of which were located at the posterior pole. This condition may be explained by the TRD at the posterior pole having relatively irregular borderlines; therefore, the cells could proliferate in multiple directions. The case with the band near the optic disc had posterior TRD. However, in another case with both a branching SRP beneath the macula and a long linear SRP at the nasal area, we found RD extending from the para-inferotemporal arcade to the nasal area without involving the macula. We presumed that in this case, the TRD involved the macula at the beginning, and the submacular fluid absorbed gradually over time for some reason, leaving only the branching SRP beneath the macula. The mechanism underlying the development of spontaneous macular reattachment in this case presumably involved the occurrence of posterior vitreous detachment (28).

Previous studies have evaluated different surgical management strategies for SRP in PVR, including PPV and scleral buckling surgery (6, 8, 9, 29, 30). Pars plana vitrectomy is needed for eyes with posterior and extensive anterior epiretinal proliferation in order to remove the contractile membranes and release the retinal traction. Lewis et al. reported that only 28% of subretinal strands require removal or transection therapy during vitrectomy and the patients who do not require removal or transection surgery have a relatively better visual prognosis (4). Many PVR patients with only SRP and no preretinal membrane can be treated successfully by scleral buckling surgery. Wallyn and Hilton reported a retinal reattachment rate of 95% with scleral buckling surgery in 20 eyes with pure SRP (13). Yao et al. reported that single scleral buckling surgery anatomical success was 90% in 40 eyes with RRD and SRP (29). Ghasemi Falavarjani et al. reported that the single surgery anatomical success rate was 88.7% in 44 eyes with RRD associated with SRP (9). Some earlier studies classified SRP following RRD into two main types (31). The first type tends to form diffuse cell sheets that do not interfere with RD, and the retina may be reattached with scleral buckling procedures alone in the absence of contractile epiretinal proliferation (32). The second type embodies taut membranes or bands, which raise the neuroretina and impede RD surgery (4). Laboratory investigations found that the first type is usually composed of glial cells and contains little or no extracellular material. Glial membranes are thin, and therefore, they rarely cause structural changes requiring surgical intervention. In contrast, the second type is composed of up to 95% RPE-derived cells, while the extracellular component includes fibrin and collagen types I to IV (31). In our study, 11 eyes (91.7%) in the study group underwent PPV without subretinal band removal or transection procedure. We only performed retinotomy and SRP removal in one eye (8.3%), since the subretinal band prevented retinal flattening after drainage of the SRF. The SRP removed from this eye contained a lot of pigment, which indicates that this SRP is more likely to be the second type. The retina was attached in all eyes after the silicon-oil-removal surgery, even in the eyes with submacular bands. This result may indicate that most SRP in PDR do not interfere with conventional retinal reattachment maneuvers. Therefore, we speculate that glial cells are the main component of most subretinal bands in PDR, which may also contain some RPE-derived cells.

Our current study, however, is limited by its retrospective nature and limited number of patients. In addition, since only one eye had undergone SRP removal and no pathological examination was performed, we cannot determine the exact pathological composition of SRP in PDR. Further evaluation and observation over a longer period as well as pathological study are required. Despite these limitations, this is the first report analyzing the clinical manifestations and surgical results of PDR patients with SRP.

In conclusion, this study showed that SRP in PDR was associated with TRD. SRP had no association with prior anti-VEGF injection or PRP. The retina could reattach successfully after PPV without removal or transection of SRP in most eyes. Moreover, the visual outcomes were generally favorable with the current treatment. Future prospective studies with longer follow-up periods are needed to confirm our findings.

## Data Availability Statement

The raw data supporting the conclusions of this article will be made available by the authors, without undue reservation.

## Author Contributions

CW and RD designed this study and wrote the article. CW, XZ, and ZC collected and analyzed the data. RD and YC critically revised the article. All authors read and approved the final version of the manuscript.

## Funding

This work was supported by the Non-profit Central Research Institute Fund of Chinese Academy of Medical Sciences (2018PT32029).

## Conflict of Interest

The authors declare that the research was conducted in the absence of any commercial or financial relationships that could be construed as a potential conflict of interest.

## Publisher's Note

All claims expressed in this article are solely those of the authors and do not necessarily represent those of their affiliated organizations, or those of the publisher, the editors and the reviewers. Any product that may be evaluated in this article, or claim that may be made by its manufacturer, is not guaranteed or endorsed by the publisher.

## References

[1] Antonetti DA Klein R Gardner TW. Diabetic retinopathy. N Engl J Med. (2012) 366:1227–39. 10.1056/NEJMra1005073 22455417

[2] Oellers P Mahmoud TH. Surgery for proliferative diabetic retinopathy: new tips and tricks. J Ophthalmic Vis Res. (2016) 11:93–9. 10.4103/2008-322X.180697 27195092 PMC4860995

[3] Kim LA Wong LL Amarnani DS Bigger-Allen AA Hu Y Marko CK. Characterization of cells from patient-derived fibrovascular membranes in proliferative diabetic retinopathy. Mol Vis. (2015) 21:673–87. 26120272 PMC4462955

[4] Lewis H Aaberg TM Abrams GW McDonald HR Williams GA Mieler WF. Subretinal membranes in proliferative vitreoretinopathy. Ophthalmology. (1989) 96:1403–14; discussion 14–5. 10.1016/S0161-6420(89)32712-6 2780008

[5] Miura M Ideta H. Factors related to subretinal proliferation in patients with primary rhegmatogenous retinal detachment. Retina. (2000) 20:465–8. 10.1097/00006982-200009000-00006 11039420

[6] Machemer R. Surgical approaches to subretinal strands. Am J Ophthalmol. (1980) 90:81–5. 10.1016/S0002-9394(14)75080-9 7395961

[7] Wilkes SR Mansour AM Green WR. Proliferative vitreoretinopathy. Histopathology of retroretinal membranes. Retina. (1987) 7:94–101. 10.1097/00006982-198700720-00007 3628996

[8] Schwartz D de la Cruz ZC Green WR Michels RG. Proliferative vitreoretinopathy Ultrastructural study of 20 retroretinal membranes removed by vitreous surgery. Retina. (1988) 8:275–81. 10.1097/00006982-198808040-00010 3231920

[9] Ghasemi Falavarjani K Alemzadeh SA Modarres M Parvaresh MM Hashemi M Naseripour M. Scleral buckling surgery for rhegmatogenous retinal detachment with subretinal proliferation. Eye (Lond). (2015) 29:509–14. 10.1038/eye.2014.341 25613841 PMC4816362

[10] Kothari N Kuriyan AE Flynn HW Jr. Spectral domain optical coherence tomography imaging of subretinal bands associated with chronic retinal detachments. Clin Ophthalmol. (2016) 10:467–70. 10.2147/OPTH.S99754 27099457 PMC4820191

[11] Holladay JT. Visual acuity measurements. J Cataract Refract Surg. (2004) 30:287–90. 10.1016/j.jcrs.2004.01.014 15030802

[12] Lange C Feltgen N Junker B Schulze-Bonsel K Bach M. Resolving the clinical acuity categories “hand motion” and “counting fingers” using the Freiburg Visual Acuity Test (FrACT). Graefes Arch Clin Exp Ophthalmol. (2009) 247:137–42. 10.1007/s00417-008-0926-0 18766368

[13] Wallyn RH Hilton GF. Subretinal fibrosis in retinal detachment. Arch Ophthalmol. (1979) 97:2128–9. 10.1001/archopht.1979.01020020446006 508180

[14] Lertsumitkul S Whitcup SM Chan CC Nussenblatt RB. Subretinal fibrosis in Vogt-Koyanagi Harada syndrome. Ophthalmology. (2001) 108:1371. 10.1016/S0161-6420(01)00665-0 11470684

[15] Steeples LR Ashworth J Jones N. Multifocal chorioretinitis with progressive subretinal fibrosis in a young child. BMJ Case Rep. (2015) 2015:bcr2015212526. 10.1136/bcr-2015-212526 26468224 PMC4612310

[16] Pitts RE Awan KJ Yanoff M. Choroidal melanoma with massive retinal fibrosis and spontaneous regression of retinal detachment. Surv Ophthalmol. (1976) 20:273–80. 10.1016/0039-6257(76)90226-5 1246673

[17] Ong SS Cummings TJ Vajzovic L Mruthyunjaya P Toth CA. Comparison of optical coherence tomography with fundus photographs, fluorescein angiography, and histopathologic analysis in assessing coats disease. JAMA Ophthalmol. (2018). 10.1001/jamaophthalmol.2018.5654 30476946 PMC6439851

[18] Fong DS Segal PP Myers F Ferris FL Hubbard LD Davis MD. Subretinal fibrosis in diabetic macular edema. ETDRS report 23 Early Treatment Diabetic Retinopathy Study Research Group. Arch Ophthalmol. (1997) 115:873–7. 10.1001/archopht.1997.01100160043006 9230827

[19] Chaikitmongkol V Bressler NM. Intraretinal fibrosis in exudative diabetic macular edema after ranibizumab treatments. Retin Cases Brief Rep. (2014) 8:336–9. 10.1097/ICB.0000000000000063 25372542

[20] Ricker LJ Kijlstra A de Jager W Liem AT Hendrikse F La Heij EC. Chemokine levels in subretinal fluid obtained during scleral buckling surgery after rhegmatogenous retinal detachment. Invest Ophthalmol Vis Sci. (2010) 51:4143–50. 10.1167/iovs.09-5057 20335622

[21] Linsenmeier RA Padnick-Silver L. Metabolic dependence of photoreceptors on the choroid in the normal and detached retina. Invest Ophthalmol Vis Sci. (2000) 41:3117–23. 10967072

[22] Khristov V Wan Q Sharma R Lotfi M Maminishkis A Bharti K. Polarized human retinal pigment epithelium exhibits distinct surface proteome on apical and basal plasma membranes. Methods Mol Biol. (2018) 1722:223–47. 10.1007/978-1-4939-7553-2_15 29264809 PMC8785602

[23] Pastor JC. de la Rua ER, Martin F. Proliferative vitreoretinopathy: risk factors and pathobiology. Prog Retin Eye Res. (2002) 21:127–44. 10.1016/S1350-9462(01)00023-4 11906814

[24] Hiscott P Morino I Alexander R Grierson I Gregor Z. Cellular components of subretinal membranes in proliferative vitreoretinopathy. Eye (Lond). (1989) 3:606–10. 10.1038/eye.1989.94 2483551

[25] Machemer R van Horn D Aaberg TM. Pigment epithelial proliferation in human retinal detachment with massive periretinal proliferation. Am J Ophthalmol. (1978) 85:181–91. 10.1016/S0002-9394(14)75946-X 623188

[26] Weller M Wiedemann P Heimann K. Proliferative vitreoretinopathy–is it anything more than wound healing at the wrong place? Int Ophthalmol. (1990) 14:105–17. 10.1007/BF00154210 2187005

[27] Laqua H Machemer R. Glial cell proliferation in retinal detachment (massive periretinal proliferation). Am J Ophthalmol. (1975) 80:602–18. 10.1016/0002-9394(75)90390-6 810029

[28] Cho HY Chung SE Kim JI Park KH Kim SK Kang SW. Spontaneous reattachment of rhegmatogenous retinal detachment. Ophthalmology. (2007) 114:581–6. 10.1016/j.ophtha.2006.05.080 17207530

[29] Yao Y Jiang L Wang ZJ Zhang MN. Scleral buckling procedures for longstanding or chronic rhegmatogenous retinal detachment with subretinal proliferation. Ophthalmology. (2006) 113:821–5. 10.1016/j.ophtha.2005.12.011 16650678

[30] Kang KT Bang SP Kim YC. Time course of subretinal proliferation after scleral buckling for rhegmatogenous retinal detachment repair. Semin Ophthalmol. (2018) 34:1–10. 10.1080/08820538.2018.1544647 30424712

[31] Hiscott P Grierson I. Subretinal membranes of proliferative vitreoretinopathy. Br J Ophthalmol. (1991) 75:53. 10.1136/bjo.75.1.53 1991089 PMC504108

[32] Federman JL Folberg R Ridley M Arbizo VA. Subretinal cellular bands. Trans Am Ophthalmol Soc. (1983) 81:172–81. 6676968 PMC1312448

